# Supplementary material for: Competition for iron shapes metabolic antagonism between Bacillus subtilis and Pseudomonas marginalis
Source: ISME J. 2024 Jan 10;18(1):wrad001. doi: 10.1093/ismejo/wrad001 (PMC10811728; doi:10.1093/ismejo/wrad001)
Supplement: Supplemental_materials_R2_wrad001 [file supplemental_materials_r2_wrad001.pdf]

Supplementary material

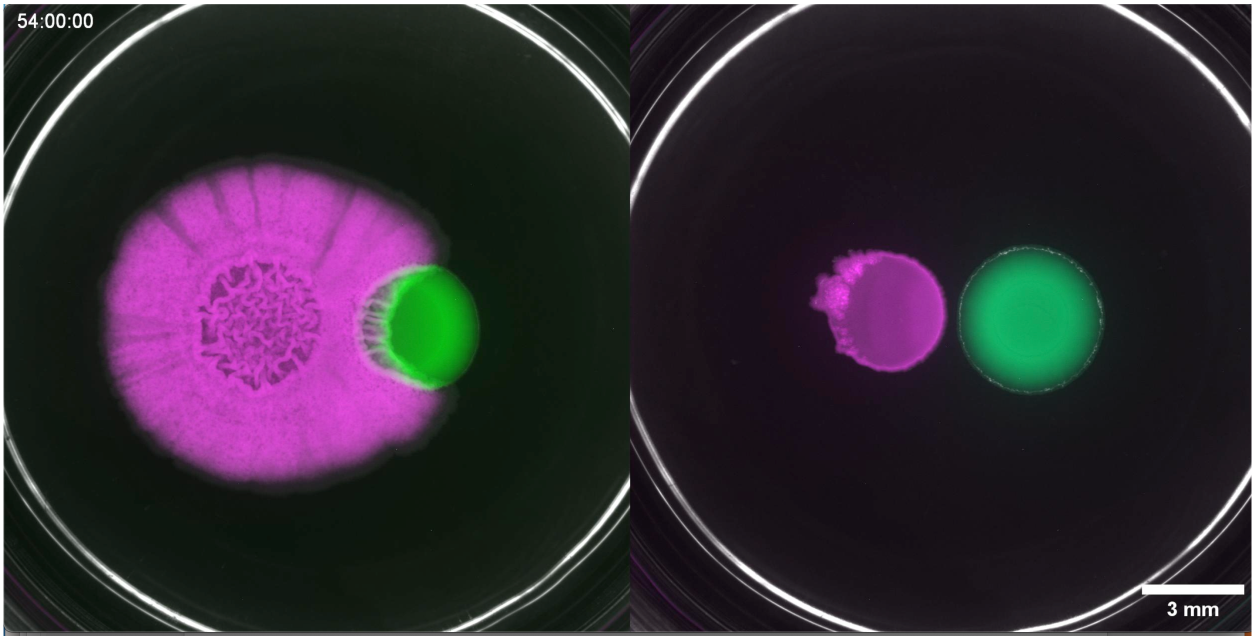

Video S1: Interaction timelapse. DK1042 (left, magenta) or Δ*dhbA* (right, magenta) were spotted next to PS92 (green) on King's B agar and incubated at 30 °C in a Zeiss Axio Zoom V16 stereomicroscope. Images were acquired every 15 minutes for 72h.

Dataset S1: Significantly differentially expressed genes from DK1042-PS92 colony cocultures.

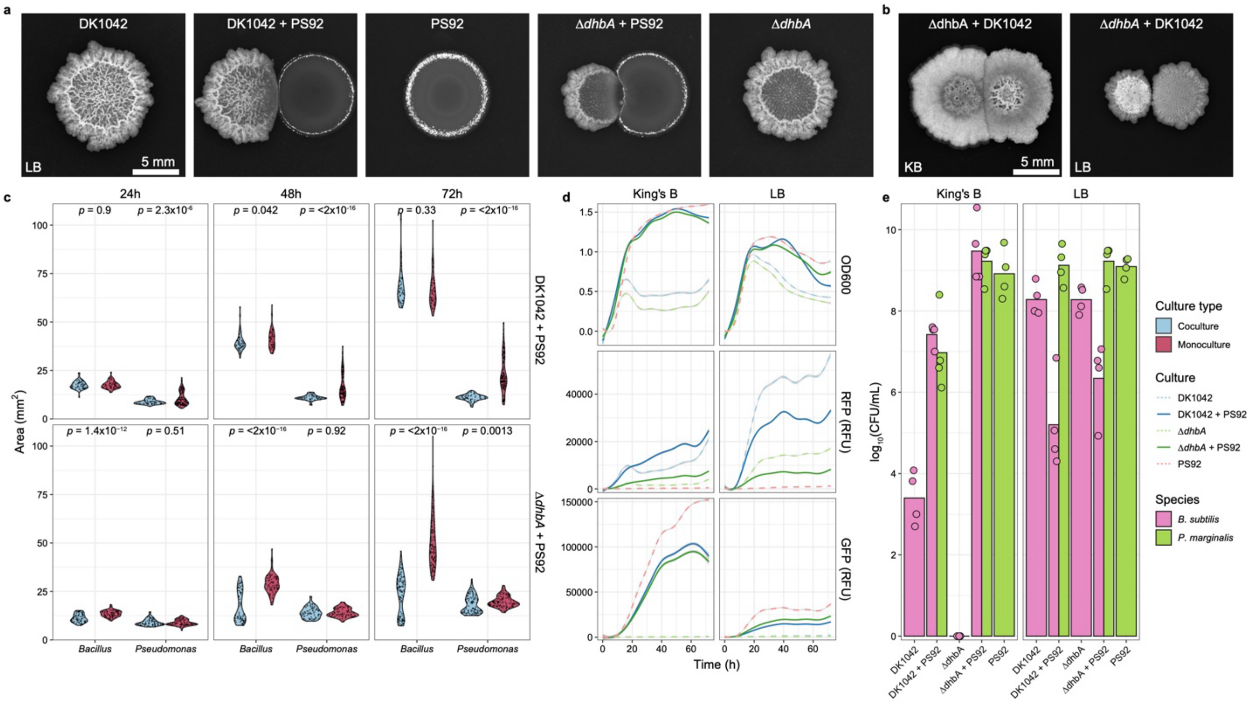

Figure S1: Interaction is constrained to solid King's B. a: Colonies grown on LB for 72h at 30 °C. **b:** Δ*dhbA* spotted next to DK1042 on King's B and LB and grown at 30 °C for 72h. **c:** Non-normalized colony-areas visualized in Figure 1. p-values are from Student's t-test adjusted for multiple testing by the Benjamini-Hochberg method. **d:** Growth in liquid King's B and LB. Strains were mixed in 96-well plates in equal starting density and growth was measured on a microplate reader with fluorescence and optical density. Starting densities were adjusted to be identical in monoculture and coculture experiments RFP: mKate2 signal (*Bacillus*), GFP: msfGFP signal (*Pseudomonas*). **e:** Viable cell count from planktonic cultures grown in Erlenmeyer flasks.

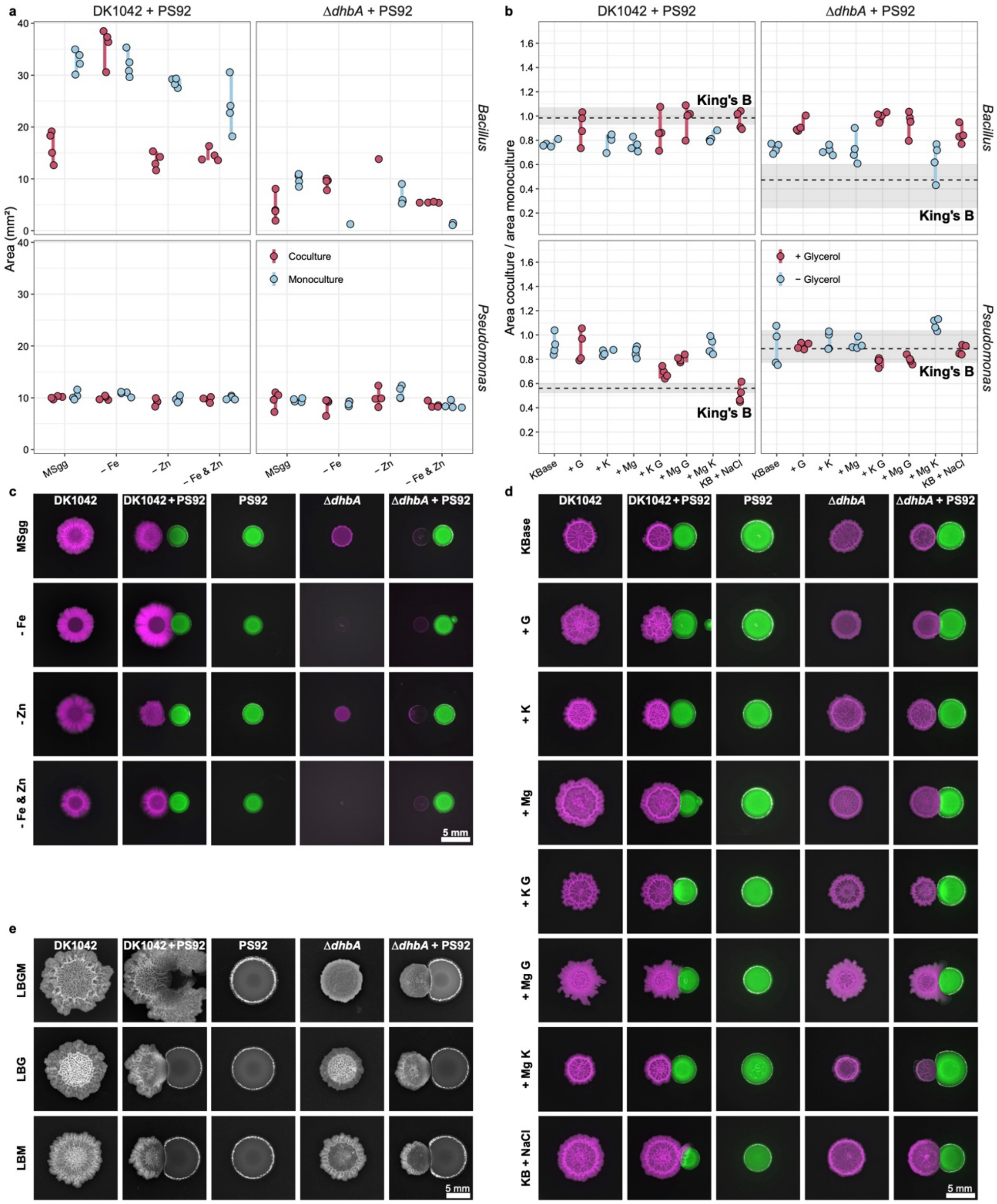

Figure S2: Interaction depends on medium composition. a: Colony area of *Bacillus* and *Pseudomonas* in interactions on MSgg with and without supplemented metals. Interactions were cultivated at 30 °C for 72h. **b:** Relative colony size of *Bacillus* and PS92 cultivated on King's B with or without supplemented Mg^{2+}, K^{+}, or glycerol (G). KBase is water and 20 g/L peptone. Dashed lines and squares are median, 1st, and 3rd quantile, respectively for DK1042 + PS92 (left) and Δ*dhbA* + PS92 (right) on regular King's B. **c and d:** Stereomicroscopy of representative samples from **a** and **b. e:** Stereomicroscopy of representative samples grown on LB with either supplemented glycerol (1% v/v, LBG), Mn^{2+} (100 µM, LBM) or both (LBGM).

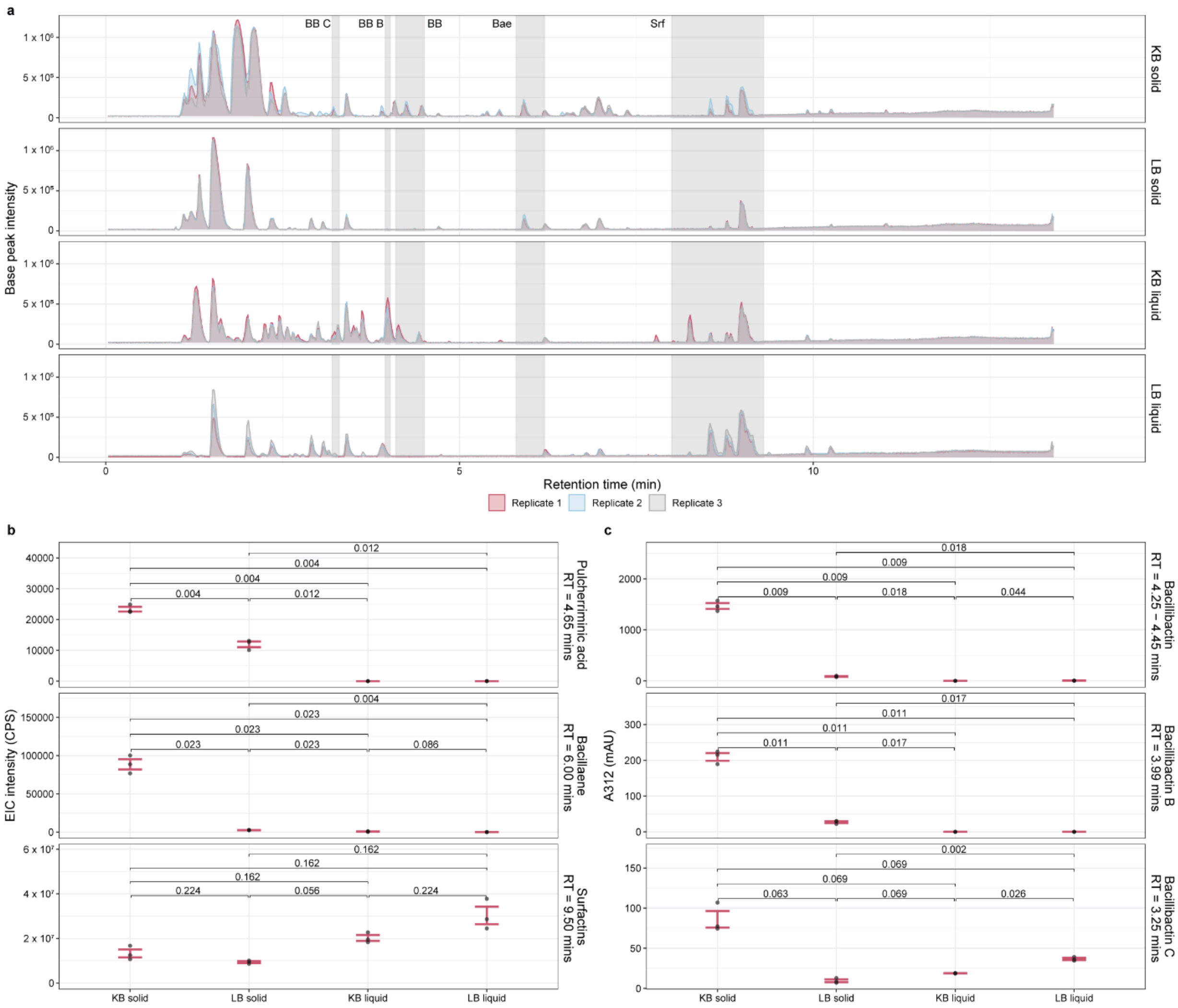

Figure S3: LC-MS in solid and liquid King's B and LB. a: Base peak chromatogram of DK1042 grown at 30 °C for 72h. Base peak intensity is measured on all eluted molecules over time. Highlights are approximate elution times for the indicated molecules (other molecules can elute at the same time point). **b:** Integrated peak area of select secondary metabolites measured in their respective ion chromatograms extracted by m/z-value. p-values are from Student's t-test adjusted for multiple testing by the Benjamini-Hochberg method. **c:** Same as **b**, but with Bacillibactins measured from their UV chromatograms. BB: Bacillibactin, Bae: Bacillaene, Srf: Surfactin.

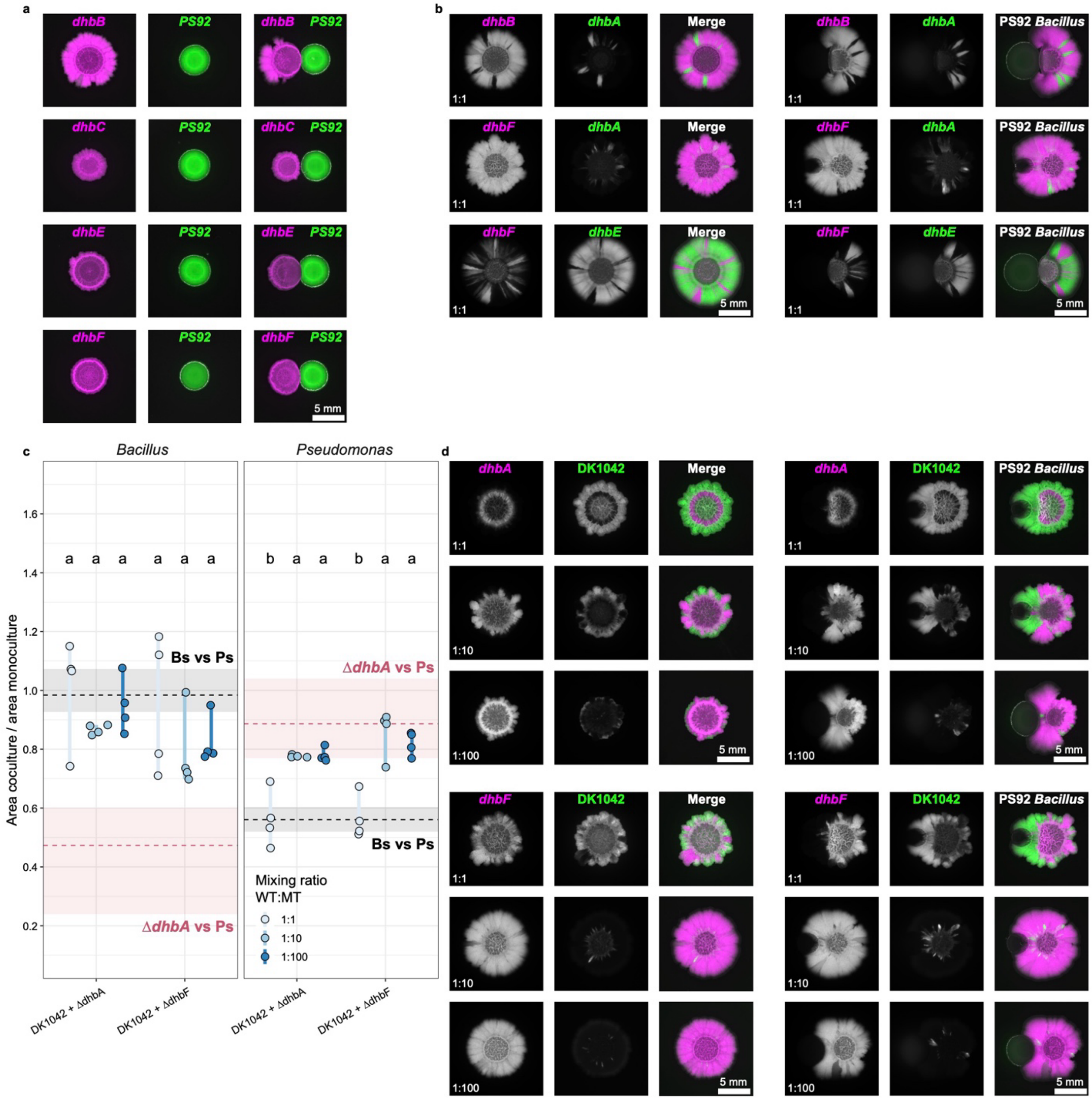

Figure S4: DK1042 can complement bacillibactin-deficient mutants. a: Stereomicroscopy of single gene deletion mutants deficient in genes encoded in the *dhb* operon cultivated on King's B at 30 °C for 72h either alone or next to PS92. See Figure 2b. **b:** Stereomicroscopy of *dhb* mutants mixed in equal ratios and their interactions as in **a**. See Figure 2c. **c:** Relative colony size of *Bacillus* and PS92 cultivated as **a** and **b**. Dashed lines and squares are median, 1st, and 3rd quantile, respectively for DK1042 + PS92 (grey) and Δ*dhbA* + PS92 (red). **d:** Stereomicroscopy of representative samples from **c**.

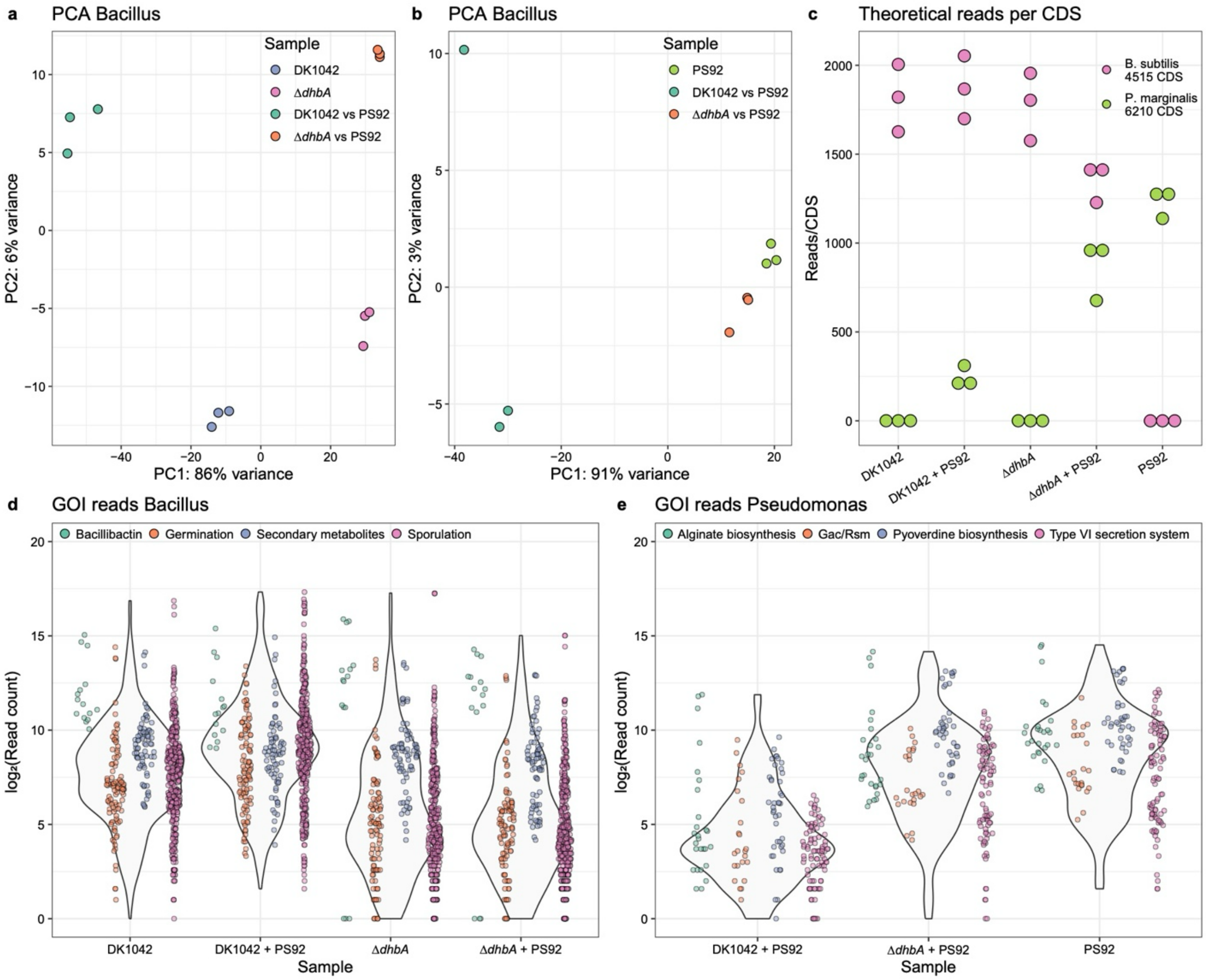

Figure S5: RNAseq quality checks. a and b: Principal component analysis of transcript reads in each sample for *Bacillus* (**a**) and *Pseudomonas* (**b**). **c:** Theoretical number of reads per coding sequence (CDS) in each genome for each sample. Calculated as n_{reads}/n_{CDS}. **d and e:** log-transformed read count for genes of interest highlighted in Figure 4a and 4b for *Bacillus* (**d**) and *Pseudomonas* (**e**).

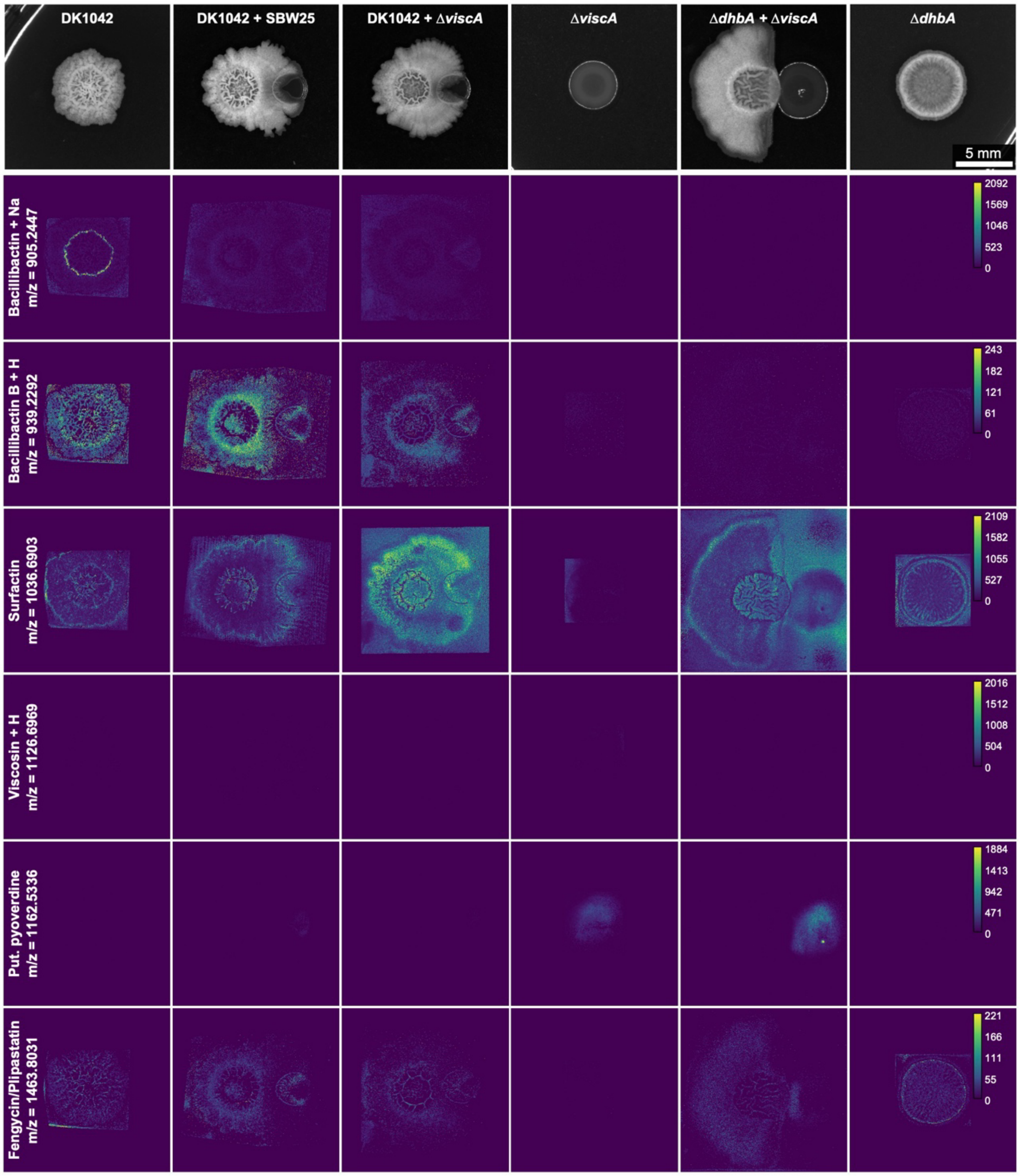

Figure S6: Viscosin is not responsible for *Bacillus* antagonism. Close-proximity colonies of DK1042 and *Pseudomonas fluorescens* SBW25 wild type and Δ*viscA* deficient in viscosin production. Colonies were cultivated on King's B at 30 °C for 72h. Mass spectrometry imaging reveals the presence/absence of select metabolites as well as their spatial localization in the interactions. Grey value is root mean squared intensity across all samples, and lookup tables were scaled identically across each metabolite. NaN pixels were set to zero. Metabolites were annotated with metaspace using the 2019 Natural Product Atlas database. m/z = 1162.5336 was manually annotated as a pyoverdine structure (this m/z-value is different from the pyoverdine structure in figure 4).

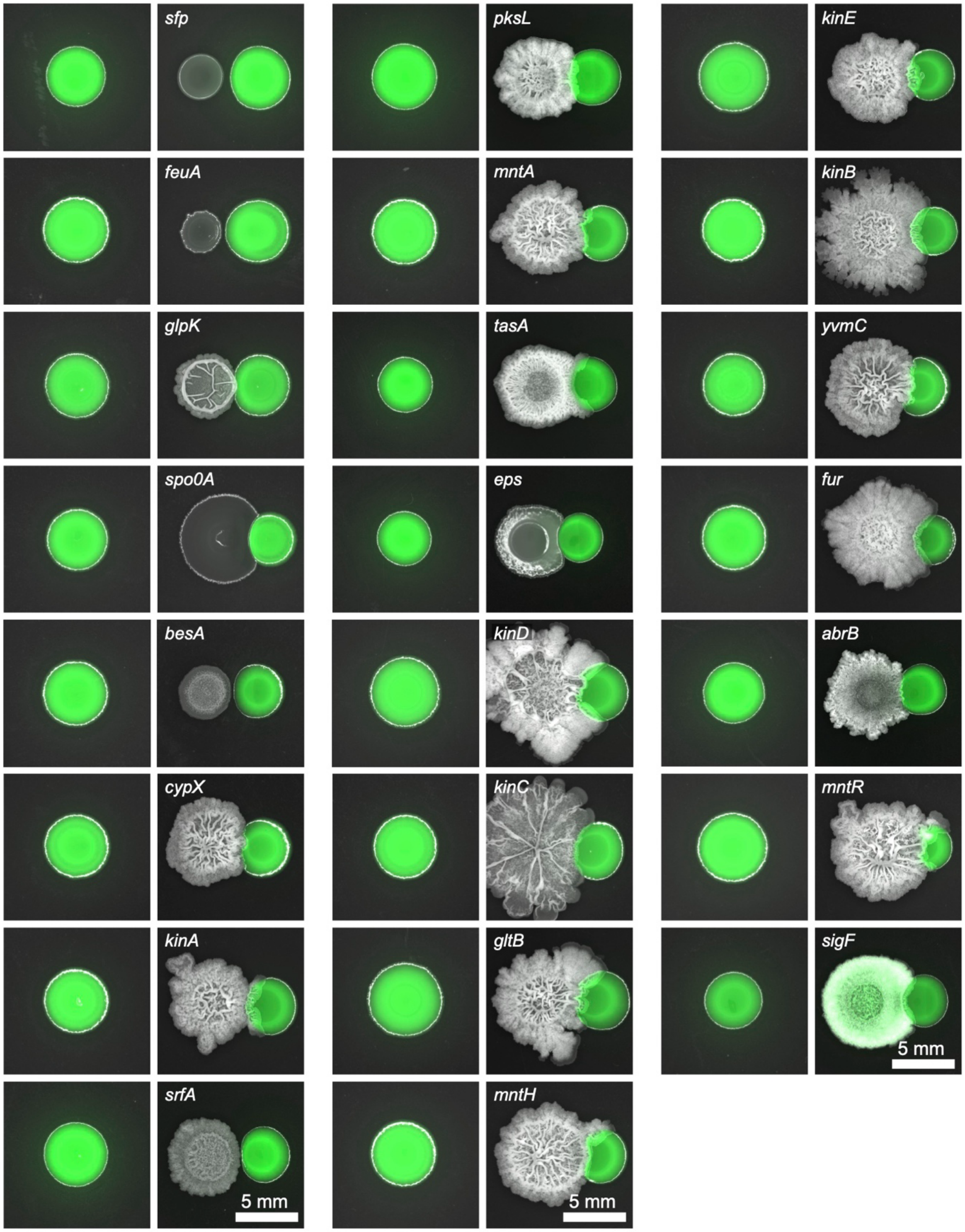

Figure S7: *Bacillus* mutant interactions with PS92. Stereomicroscopy of representative mutant interactions from Figure 5. Green is PS92.

Supplementary methods

Culturing

Growth was assessed by inoculating DK1042 or PS92 into the wells of a 96-well microtiter plate at a final OD_{600} = 0.001 and incubating the plate in a Synergy HTX multi-mode reader at 30 ℃ with shaking capturing optical density at 600 nm (OD_{600}), msfGFP (Ex: 485/20 nm, Em: 528/20 nm), and mKate2 (Ex: 590/20 nm, Em: 635/32 nm) every 15 minutes.

Colony forming units from liquid cultures were counted from cultures grown in 50 mL broth in 250 mL Erlenmeyer flasks. 1 mL culture was harvested from each sample, serially diluted, and spread on selective media. Four independent cultures were used as biological replicates, and three 1 mL volumes were taken from each flask as technical replicates.

Iron limitation was achieved by adding 2,2-bypridine or by not adding $FeCl_2$ to the media (e.g. with MSgg). Limitation of zinc in MSgg, was achieved by not adding $ZnCl_2$. KBase consisted of 20 g/L peptone in deionized H_2O. Glycerol, K_2HPO_4, $MgSO_4 \cdot 7H_2O$, and NaCl was added into the media before autoclavation, in the concentrations specified for KB (or 5 g/L in the case of NaCl).

.

Pairwise interactions on agar

DK1042 and PS92 were routinely spotted 5 mm apart on agar surfaces using 2 µL overnight culture adjusted to an optical density at 600 nm (OD_{600}) of 1.0 in appropriate media. Prior to spotting, plates were dried for 30 min in a lateral flow hood and incubated at 30°C. This experimental setup was employed for all coculture colony assays with multiple types of media and strains. When spotting mixed cultures, each species was adjusted to an OD_{600} of 1.0 and the two strains were then mixed in equal volumes. For mixed *B. subtilis* cultures, wild-type (WT) and mutant strains were mixed 1:1, 1:10, or 1:100, based on OD_{600}, before spotting 2 µL as detailed above.

Timelapse microscopy

Timelapse series were acquired with the Carl Zeiss Axiozoom V.16 stereomicroscope (see main text) at 15-minute intervals for 72 hours. Bacterial cultures were spotted on agar solidified in a 35 mm petri dish which was then placed into a Tokai Hit stage top incubator pre-warmed to 30 °C. Sterile MilliQ water was added to the incubator water bath to maintain constant humidity in the chamber.

Genome assembly

Genomes were assembled using Trycycler (V0.5.3) [1] when long read sequencing depth was adequate for subsetting or Unicycler (V0.5.0) [2] in all other cases. Adapters were removed using Porechop (0.2.4 https://github.com/rrwick/Porechop), and reads were filtered with Filtlong (V0.2.1 https://github.com/rrwick/Filtlong) to remove the 5% with the lowest quality score and any read shorter than 1kb. Following the Trycycler standard protocol, the filtered long read set is subset into 12 equal sets which are then assembled with Flye (2.9-b1768)[3], Miniasm (0.3-r179)[4] along with Minipolish (v0.1.2)[5], and Raven (1.7.0)[6]. From these 12 sub-assemblies,

Trycycler creates a consensus using MUSCLE (v3.8.1551)[7] that is polished with MEDAKA (1.5.0 https://github.com/nanoporetech/medaka) using the long reads. The short reads are then similarly trimmed and filtered using FastP (0.12.4)[8] and subsequently aligned to the long-read consensus using bwa (0.7.17-r1188)[9]. The aligned short reads are then used by Polypolish (V0.5.0)[10] and Polca.sh from Masurca (4.0.5)[11] in multiple rounds of polishing to yield the final single-contig assembly. Completeness and contamination were assessed using CheckM (V1.1.3) [12] and whole-genome taxonomy was determined with AutoMLST [13] and the TYGS database (V342) [14].

Transcriptomics
Samples were bead-beaten at 4500 RPM in a FastPrep for 30 seconds, and otherwise subjected to the manufacturer's protocol. DNA was digested with TURBO DNase following the protocol for rigorous DNA digestion. RNA concentration was assessed on a Qubit fluorometer using the Qubit High Sensitivity kit (concentrations ranging from 20-100 ng μL^{-1}), and RNA integrity was assessed on an Agilent Bioanalyzer 2100 using Agilent RNA 6000 nano kit. All samples had an RNA integrity number (RIN) > 7.2.

References for Supplementary materials

1. Wick RR, Judd LM, Cerdeira LT, Hawkey J, Méric G, Vezina B, et al. Trycycler: consensus long-read assemblies for bacterial genomes. *Genome Biol* 2021; **22**: 266.

2. Wick RR, Judd LM, Gorrie CL, Holt KE. Unicycler: Resolving bacterial genome assemblies from short and long sequencing reads. *PLoS Comput Biol* 2017; **13**: e1005595.

3. Kolmogorov M, Yuan J, Lin Y, Pevzner PA. Assembly of long, error-prone reads using repeat graphs. *Nat Biotechnol* 2019; **37**: 540–546.

4. Li H. Minimap and miniasm: Fast mapping and de novo assembly for noisy long sequences. *Bioinformatics* 2016; **32**: 2103–2110.

5. Wick RR, Holt KE. Benchmarking of long-read assemblers for prokaryote whole genome sequencing. *F1000Res* 2019; **8**: 2138.

6. Vaser R, Šikić M. Time- and memory-efficient genome assembly with Raven. *Nat Comput Sci* 2021; **1**: 332–336.

7. Edgar RC. MUSCLE: Multiple sequence alignment with high accuracy and high throughput. *Nucleic Acids Res* 2004; **32**: 1792–1797.

8. Chen S, Zhou Y, Chen Y, Gu J. Fastp: An ultra-fast all-in-one FASTQ preprocessor. *Bioinformatics* 2018; **34**: i884–i890.

9. Li H, Durbin R. Fast and accurate short read alignment with Burrows-Wheeler transform. *Bioinformatics* 2009; **25**: 1754–1760.

10. Wick RR, Holt KE. Polypolish: Short-read polishing of long-read bacterial genome assemblies. *PLoS Comput Biol* 2022; **18**: e1009802.

11. Zimin A v., Salzberg SL. The genome polishing tool POLCA makes fast and accurate corrections in genome assemblies. *PLoS Comput Biol* 2020; **16**: e1007981.

12. Parks DH, Imelfort M, Skennerton CT, Hugenholtz P, Tyson GW. CheckM: Assessing the quality of microbial genomes recovered from isolates, single cells, and metagenomes. *Genome Res* 2015; **25**: 1043–1055.

13. Alanjary M, Steinke K, Ziemert N. AutoMLST: An automated web server for generating multi-locus species trees highlighting natural product potential. *Nucleic Acids Res* 2019; **47**: W276–W282.

14. Meier-Kolthoff JP, Göker M. TYGS is an automated high-throughput platform for state-of-the-art genome-based taxonomy. *Nat Commun* 2019; **10**: 2182.

**Strain**			
Bacillus subtilis	**Genotype**		**Ref**
B. subtilis DK1042	3610 $comI^{Q12I}$		[1]
TB501.1	3610 $comI^{Q12I}$ *amyE*::$P_{hyperspank}$-mKate2-$Spec^R$		[2]
TB500.1	3610 $comI^{Q12I}$ *amyE*::$P_{hyperspank}$-eGFP-$Spec^R$		[2]
Δ*dhbA*	DK1042 *dhbA*::Kan^R		This study
Δ*dhbA* mKate2	DK1042 *dhbA*::Kan^R *amyE*::$P_{hyperspank}$-mKate2-$Spec^R$		This study
Δ*dhbA* eGFP	DK1042 *dhbA*::Kan^R *amyE*::$P_{hyperspank}$-eGFP-$Spec^R$		This study
Δ*dhbB* mKate2	DK1042 *dhbB*::Kan^R *amyE*::$P_{hyperspank}$-mKate2-$Spec^R$		This study
Δ*dhbC* mKate2	DK1042 *dhbC*::Kan^R *amyE*::$P_{hyperspank}$-mKate2-$Spec^R$		This study
Δ*dhbE* mKate2	DK1042 *dhbE*::Kan^R *amyE*::$P_{hyperspank}$-mKate2-$Spec^R$		This study
Δ*dhbE* eGFP	DK1042 *dhbE*::Kan^R *amyE*::$P_{hyperspank}$-eGFP-$Spec^R$		This study
Δ*dhbF* mKate2	DK1042 *dhbF*::Kan^R *amyE*::$P_{hyperspank}$-mKate2-$Spec^R$		This study
DS3337	3610 Δ*sfp::*MLS^R		[3]
Δ*mntH*	DK1042 *mntH*::Kan^R		This study
Δ*mntA*	DK1042 *mntA*::Kan^R		This study
Δ*mntR*	DK1042 *mntR*::Kan^R		This study
DS4085	3610 *pksL*::Cm^R		[4]
DS1122	3610 Δ*srfA-C*::Tn10-$Spec^R$		[5]
Δ*gltB*	DK1042 *gltB*::Kan^R		This study
Δ*glpK*	DK1042 *glpK*::Kan^R		This study
TB863	3610 Δ*tasA::*Km^R		[2]
Δ*abrB*	DK1042 *abrB*::Kan^R		This study
Δ*cypX*	DK1042 *cypX*::Kan^R		This study
TB601	3610 Δ*epsA-O::*Tet^R		[2]
Δ*yvmC*	DK1042 *yvmC*::Kan^R		This study
TB398	DK1042 *kinA*::Erm^R		This study
TB399	DK1042 *kinB*::Tet^R		This study
TB400	DK1042 *kinC*::$Spec^R$		This study
TB401	DK1042 *kinD*::Cm^R		This study
TB402	DK1042 *kinE*::Cm^R		This study
Δ*abrB*	DK1042 *abrB*::kan^R		This study
Δ*spo0A*	DK1042 *spo0A*::Kan^R		This study
Δ*fur*	DK1042 *fur*::Kan^R		This study
Δ*feuA*	DK1042 *feuA*::Kan^R		This study
Δ*feuA* mKate2	DK1042 *feuA*::Kan^R *amyE*::$P_{hyperspank}$-mKate2-$Spec^R$		This study
Δ*besA*	DK1042 *besA*::Kan^R		This study
Δ*besA* mKate2	DK1042 *besA*::Kan^R *amyE*::$P_{hyperspank}$-mKate2-$Spec^R$		This study
Pseudomonas	**Genotype**	**Genome Accession**	**Ref**
P. marginalis PS92	WT isolate	CP125381	This study
P. marginalis PS92 msfGFP	PS92 attTn7::P_{14g}-msfGFP-Gm^R		This study
Pseudomonas sp. P5_109	WT isolate	CP125380	This study
Pseudomonas sp. P5_152	WT isolate	JASFAH000000000	This study
P. zeae P8_72	WT isolate	JASFAG000000000	This study
Pseudomonas sp. P8_139	WT isolate	CP125379	This study
Pseudomonas sp. P8_229	WT isolate	CP125378	This study
Pseudomonas sp. P8_241	WT isolate	CP125377	This study
Pseudomonas sp. P8_250	WT isolate	JASFAF000000000	This study
Pseudomonas sp. P9_31	WT isolate	CP125375	This study
Pseudomonas sp. P9_2	WT isolate	CP125376	This study
Pseudomonas sp. P9_32	WT isolate	CP125374	This study
Pseudomonas sp. P9_35	WT isolate	CP125373	This study
P. germanicum P9_87	WT isolate	CP125372	This study
P. protegens P9_191	WT isolate	JASFAE000000000	This study
Escherichia coli	**Genotype**		**Ref**

E. coli CC118	CC118 λpir/pBG42	[6]
E. coli HB101	HB101 /pRK600	[6]
E. coli CC118	CC118 λpir/pTNS2	[6]

**Oligo ID**	**Description**	**Sequence (5' – 3')**
oTB134	sfp up	GGTGTCAAGCTGTTGATGAG
oTB135	sfp down	AAGCATCTCCGCCTGTACAC
oTB249	feuA up	GCTGAGATGCTTTGCCCCAT
oTB250	feuA down	CGAACGCATCGTTCAGCAAA
oTB251	fur up	GCATCGACAGCTGTTTTCAG
oTB252	fur down	GGAACTCTGCGCGTATTTTGT
oTB232	dhbA up	CGATGAGGAGACGCTAAG
oTB233	dhbA down	CGCTGGATGTCTGTTTGG
oTB255	dhbB up	GTGCCGCAAAGCTTCTTCAA
oTB256	dhbB down	CTGTCCATGATGCGGCAATG
oTB257	dhbC up	AGCCCTTGTGTCAAGCTCTC
oTB258	dhbC down	CCCGGATCAACGGAAACAGA
oTB259	dhbE up	GATTCGGATCAGGCACCCAT
oTB260	dhbE down	GGTTCAAAGCCTGAGGACGA
oTB261	dhbF up	GTGGGCCGCTGTGTTTTTAG
	dhbF down	GCAGAGGTGACTTTCGTGGA
	abrB up	CGCAATGTACCAAAGCGTGA
	abrB down	GCTATGAAGGTAAGGATTTTGTCG
	glpK up	CTGCCAAGCTGGGTGTTTTC
	glpK down	CCGAATGTGCCGCCTTTATG
	gltB up	CGGCCTGTAATTGACCGGAT
	gltB down	CGCAAGCATCGAAGAGCAAA
	mntH up	AGCAGAACCGACCAAGAAGG
	mntH down	GACTTCTTGGCACATGGGGA
	mntA up	GGCTGATTTGCCTTTTCGCA
	mntA down	ACAGCCTGGTTTAGGGGTTG
	mntR up	GACCCTTTCTGCAAATCGCC
	mntR down	CGGATGCAGCGCAATTTCAA
	yvmC up	GATCCAGTGCGTCACCGATA
	yvmC down	TGACGTTTGGAAGGAAAAGGGA

TB398-TB402 were created by transforming DK1042 with genomic DNA from JH12638 (*kinA*), JH19980 (*kinB*) from Wang *et al.* 1997 (Genes Dev), and BAL393 (*kinC*), BAL691 (*kinD*), and BAL692 (*kinE*) from Grau *et al.* 2015 (mBio).

[1] Konkol MA, Blair KM, Kearns DB. Plasmid-encoded ComI inhibits competence in the ancestral 3610 strain of *Bacillus subtilis*. *J Bacteriol* 2013; **195:** 4085-4093.

[2] Dragoš A, Kiesewalter H, Martin M, Hsu CY, Hartmann R, Wechsler T, Eriksen C, Brix S, Drescher K, Stanley-Wall N, Kümmerli R, Kovács ÁT. Division of labor during biofilm matrix production. *Curr Biol* 2018; **28:** 1903-1913.

[3] Patrick JE, Kearns DB. Laboratory strains of *Bacillus subtilis* do not exhibit swarming motility. *J Bacteriol* 2009; **191:** 7129–7133.

[4] Müller S, Strack SN, Hoefler BC, Straight PD, Kearns DB, Kirby JR. Bacillaene and sporulation protect *Bacillus subtilis* from predation by *Myxococcus xanthus*. *Appl Environ Microbiol* 2014; **80:** 5603–5610.

[5] Chen R, Guttenplan SB, Blair KM, Kearns DB. Role of the σ^D-dependent autolysins in *Bacillus subtilis* population heterogeneity. J Bacteriol 2009; **191:** 5775–5784.
